# Qualitative differences in cellular immunogenicity elicited by hepatitis C virus T-Cell vaccines employing prime-boost regimens

**DOI:** 10.1371/journal.pone.0181578

**Published:** 2017-07-21

**Authors:** Wendy G. Tan, Iryna Zubkova, Alla Kachko, Frances Wells, Heiko Adler, Gerd Sutter, Marian E. Major

**Affiliations:** 1 Laboratory of Hepatitis Viruses, Division of Viral Products, Center for Biologics Evaluation and Research, Food and Drug Administration, Silver Spring, MD United States of America; 2 Comprehensive Pneumology Center, Research Unit Lung Repair and Regeneration, Helmholtz Zentrum München—German Research Center for Environmental Health (GmbH), Member of the German Center of Lung Research (DZL), Munich, Germany; 3 Institute for Infectious Diseases and Zoonoses, LMU University of Munich, Munich, Germany; Saint Louis University, UNITED STATES

## Abstract

T-cell based vaccines have been considered as attractive candidates for prevention of hepatitis C virus (HCV) infections. In this study we compared the magnitude and phenotypic characteristics of CD8+ T-cells induced by three commonly used viral vectors, Adenovirus-5 (Ad5), Vaccinia virus (VV) and Modified Vaccinia Ankara (MVA) expressing the HCV NS3/4A protein. C57/BL6 mice were primed with DNA expressing NS3/4A and boosted with each of the viral vectors in individual groups of mice. We then tracked the vaccine-induced CD8+ T-cell responses using pentamer binding and cytokine production analysis. Overall, our data indicate that the memory cells induced by Ad5 were inferior to those induced by VV or MVA. We found that Ad5 boosting resulted in rapid expansion and significantly higher frequencies of NS3-specific T-cells compared to VV and MVA boosting. However, the functional profiles, assessed through analysis of the memory cell marker CD127 and the anti-apoptotic molecule Bcl-2 in the blood, spleen, and liver; and measurements of interferon-gamma, tumor necrosis factor-alpha, and interleukin-2 production indicated significantly lower frequencies of long-lived memory T-cells following Ad5 boosting compared to VV and MVA. This same set of analyses suggested that the memory cells induced following boosting with MVA were superior to those induced by both Ad5 and VV. This superiority of the MVA-induced CD8+ T-cells was confirmed following surrogate challenge of mice with a recombinant mouse herpes virus expressing the HCV NS3 protein. Higher levels of NS3-specific CD8+ T-cells displaying the functional markers CD69, Ki67 and Granzyme B were found in the spleens of mice boosted with MVA compared to VV and Ad5, both alone and in combination. These data suggest that MVA may be a more successful viral vector for induction of effective CD8+ T-cell responses against hepatitis C virus.

## Introduction

Hepatitis C virus (HCV) infection is a global health threat. About 180 million people worldwide are chronically infected, with about 500,000 HCV-related deaths each year [1, 2]. Current drug therapies can clear the majority of HCV infections [3], but treatment success can be limited by numerous factors including access to care, cost of therapy, patient adherence, relative efficacy of different regimens, side effects, viral genotype and host factors. It is also unclear if individuals are protected from reinfection following drug treatment. Drug treatment of acute phase HCV infections has been shown to result in functional CD4+ and CD8+ T-cell responses [4], however, such responses have not been shown in patients successfully treated during the chronic phase [5]. Therefore, a prophylactic vaccine is still needed to prevent HCV infections across the globe.

A large body of evidence has shown that cellular immunity plays a major role in controlling acute HCV infections [6–12]. Several studies have reported that broad, polyclonal CD4+ and CD8+ T-cell responses are present in patients with self-resolved infections [8–14] and chimpanzee studies have shown that T-cells play a pivotal role during secondary exposure after spontaneous clearance and in protection from persistent infection [15–17]. For these reasons T-cell-based vaccines for HCV are highly attractive and represent an important and rapidly developing class of vaccines as prophylaxis for prevention and control of several chronic diseases such as HCV, HIV, tuberculosis and Malaria.

Successful T-cell immunity requires long-term immunological memory that can be rapidly reactivated to substantially reduce the viral loads and prevent the risk of developing chronic infection upon re-exposure. The HCV T-cell based vaccine studies reported thus far confirm that a vaccine-induced T-cell response can contribute significantly to the control of virus replication but persistent infections have frequently been seen in immunized chimpanzees following virus challenge [18] with a possibility of immune escape from the vaccine-induced immune responses. We have previously shown that an ineffective T-cell vaccine against HCV can create greater pressure for viral mutation and therefore immune escape, which may lead to persistence despite initial control of the virus [19]. We subsequently showed that memory T-cell responses leading to clearance of HCV are phenotypically different from those that result in persistence of the virus [20] suggesting that the magnitude of the response is less important than the functional quality of the induced T-cells.

The HCV-NS3 protein has been shown to be highly immunogenic, inducing a diverse repertoire of cell-mediated immune responses, and the importance of T-cells directed to this antigen for controlling viral replication have been widely reported [6, 21, 22]. In these studies we wished to determine if different viral vectors induce qualitatively different T-cell responses against the HCV NS3 antigen which may be helpful in predicting the optimal immunization method for an HCV vaccine. Studies on HCV vaccines are largely hampered by the lack of a small animal model. Chimpanzees remain the only animal model for this virus where the outcome of infection in the presence of memory immune response can be established [23], however, the availability of these animals for biomedical research has become limited. Therefore, we sought to use a mouse model to evaluate phenotypic differences between T-cells induced by different viral vectors following priming with DNA. The DNA prime/viral vector boost approach has been used in many experimental HCV T-cell vaccines in the past, most of which have included the HCV NS3 protein as a target for immune responses with varying outcomes [24]. We primed mice with plasmid DNA containing the full-length HCV-NS3/4A transgene followed by boosting with either Adenovirus 5 (Ad5), Vaccinia virus (VV) or Modified Vaccinia Ankara (MVA)-HCV-NS3/4A vectors. The correlates of protection after T-cell based vaccination are complicated and differ with each pathogenic antigen. However, certain canonical biomarkers and functional qualities expressed by antigen-experienced T-cells in the memory stage may present clues to the qualitative differences among various vaccine platforms [25, 26]. Therefore, we assessed the vaccine-induced HCV-NS3-specific CD8+ T-cells by pentamer binding assays, characterized memory phenotypic markers of the pentamer positive CD8+ T-cells and assayed for IFN-γ, TNF-alpha and IL-2 cytokine production profiles. We also assessed the phenotypes of the recall response to NS3-specific T-cells following surrogate challenge with mouse herpes virus expressing the HCV NS3 protein.

## Materials and methods

### Viral vectors and MHV-68-NS3 construction

Plasmid DNA containing the entire sequence of the HCV NS3/4A genes (Genotype 1a, strain H77) was produced as previously described [19]. Modified Vaccinia Ankara-NS3 (MVA-NS3), [27], Vaccinia virus (WR)-NS3 (VV-NS3) [28] and Adenovirus 5-NS3 (Ad5-NS3) [19] constructs were generated as previously described. MHV-68-NS3 for challenge was characterized and reconstituted as previously described [27].

### Mouse studies

All experiments were approved by the Intramural Animal Care and Use Committee of the Center for Biologics Evaluation and Research, Food and Drug Administration and carried out in strict adherence to the approved protocol, including efforts to minimize suffering of study animals. Mice were housed and maintained according to NIH Animal Research Advisory Committee guidelines. For intramuscular inoculations animals were anesthetized using 3% isoflurane inhalation. For tissue isolation animals were euthanized with carbon dioxide inhalation in a euthanasia chamber. Female C57BL/6J (B6) mice were obtained from Jackson Laboratories (Bar Harbor, ME) at 6–8 weeks old and housed in the US-FDA animal research facility.

### Primary response for identification of T-cell epitopes

B6 mice were immunized with 2x10^6^ pfu/mouse of VV-NS3 via intraperitoneal injections or 10^9^ viral particles (vp)/mouse of Ad5-NS3 delivered intramuscularly. Control mice were inoculated with Null-VV or Null-Ad5 (empty Ad5 or VV vectors).

### Prime-boost

Plasmid DNA expressing the HCV NS3/4A protein under the control of a CMV promoter was administered at 100 μg/mouse delivered via intramuscular injections to the hind quadriceps of B6 mice. At day 85 post prime, groups of mice were boosted via intra-peritoneal injections with 2x10^6^ pfu/mouse of VV-NS3 (VV), 10^8^ infectious units (IU) of MVA-NS3 (MVA) or via intramuscular injections with 10^9^ vp of Ad5-NS3 (Ad5).

### Direct challenge

B6 memory mice (85 days post boost) were challenged with 10^5^ pfu/mouse of MHV-68-NS3 live virus via intraperitoneal injections. Mice were sacrificed at day 2, 6, 9, 13 and 16 post challenge to harvest spleens and blood for proliferation studies. Virus titers were assessed on D2, D4, D6, D9, D12 and D14 by plaque assay as previously described [29].

### In vivo assays and cell isolation

Lymphocytes from the spleens, livers, and blood were isolated to assess kinetics of antigen-specific CD8+ T-cells. Spleens were homogenized into single cell suspension and red blood cells were lysed by using ACK lysing buffer (Life Technologies, Grand Island, NY) according to the manufacturer’s protocol. Splenocytes were then washed twice with RPMI supplemented with 5% FBS, penicillin-streptomycin and L-glutamine (R-5). Finally, the splenocytes were resuspended in RPMI supplemented with 10% FBS (R-10). Livers were perfused with 20ml PBS during harvest and collected in RPMI + 5% FBS. The livers were homogenized to single cell suspension, washed and resuspended in 44% Percoll (R10 + Percoll) and then underlaid with 67% Percoll (1X PBS + Percoll). The Percoll gradient was centrifuged at 2000 rpm (400 x g) for 20 minutes at room temperature (RT) with no brake. After centrifugation, the interface containing the lymphocytes was collected and washed twice with R5 and resuspended in an appropriate volume of the R10 medium to attain 1–2 million cells/mL. Blood lymphocytes were isolated by underlaying with 2 mL of Histopaque (Sigma, St Louis, MO) and centrifuged at 2000 rpm at RT for 20 minutes. The interface was collected and washed twice with R5 and then resuspended in an appropriate volume of R10 medium.

### Antibodies, pentamer staining and flow cytometry

MHC class I H2-D^b^ pentamer with the sequence GAVQNETVL (GAVQ) was purchased from ProImmune (Oxford, UK) and stained according to the manufacturer’s recommendation. Splenocytes were stimulated with NS3 peptide pools (18-mers overlapping by 11 amino-acids, NIH AIDS Reagent Program) or with individual peptides representing the various epitopes (Mimotopes, Australia). Cells were stimulated in 96 well round bottom plates with 1.5 million cells/well and 0.5μg of peptide supplemented with 2 μL Brefeldin and 2 μL Monensin per mL of R10 and incubated for 5 hours at 37°C before performing intracellular cytokine staining with antibodies. Surface staining of fluorescence-conjugated antibodies was performed according to the manufacturer’s protocol or titration. For intracellular staining, cells were permeabilized with cytoperm/cytofix (Becton Dickinson, Franklin Lakes, NJ) by following the manufacturer’s protocol before staining with fluorescence-conjugated antibodies. Cellular events were acquired using a FACS Canto II (BD Biosciences, San Jose, CA) multi-parameter flow cytometer. The following antibodies were used: Bcl-2, Ki67, CD8, CD19, CD44, Ly6C, CD62L, CD27, IFN-γ, TNF-alpha, IL-2, CD107a/b, CD4 (BD Biosciences, San Jose, CA), CD127 (E-Bioscience, San Diego, CA), CD43 (1B11), CD3, CD69 (Biolegend, San Diego, CA), fixable live-dead near IR (Life Technologies, Grand Island, NY).

### Data and statistical analyses

Flow data were analyzed with Flowjo software (Treestar, Ashford, OR) and graphical representations generated by Graphpad Prism V5 (Graphpad, San Diego, CA). Statistical analyses were performed by using a one-way analysis of variance (ANOVA) in Graphpad software to compare means for T-cell responses. When a statistically significant difference between the means was determined using one-way ANOVA a Bonferroni post hoc analysis was used to assess differences between the means of each immunized group. A p value of < 0.05 was considered significant.

## Results

### Identification of novel H2-Db CD8+ T-cell epitopes

**Fig 1:**
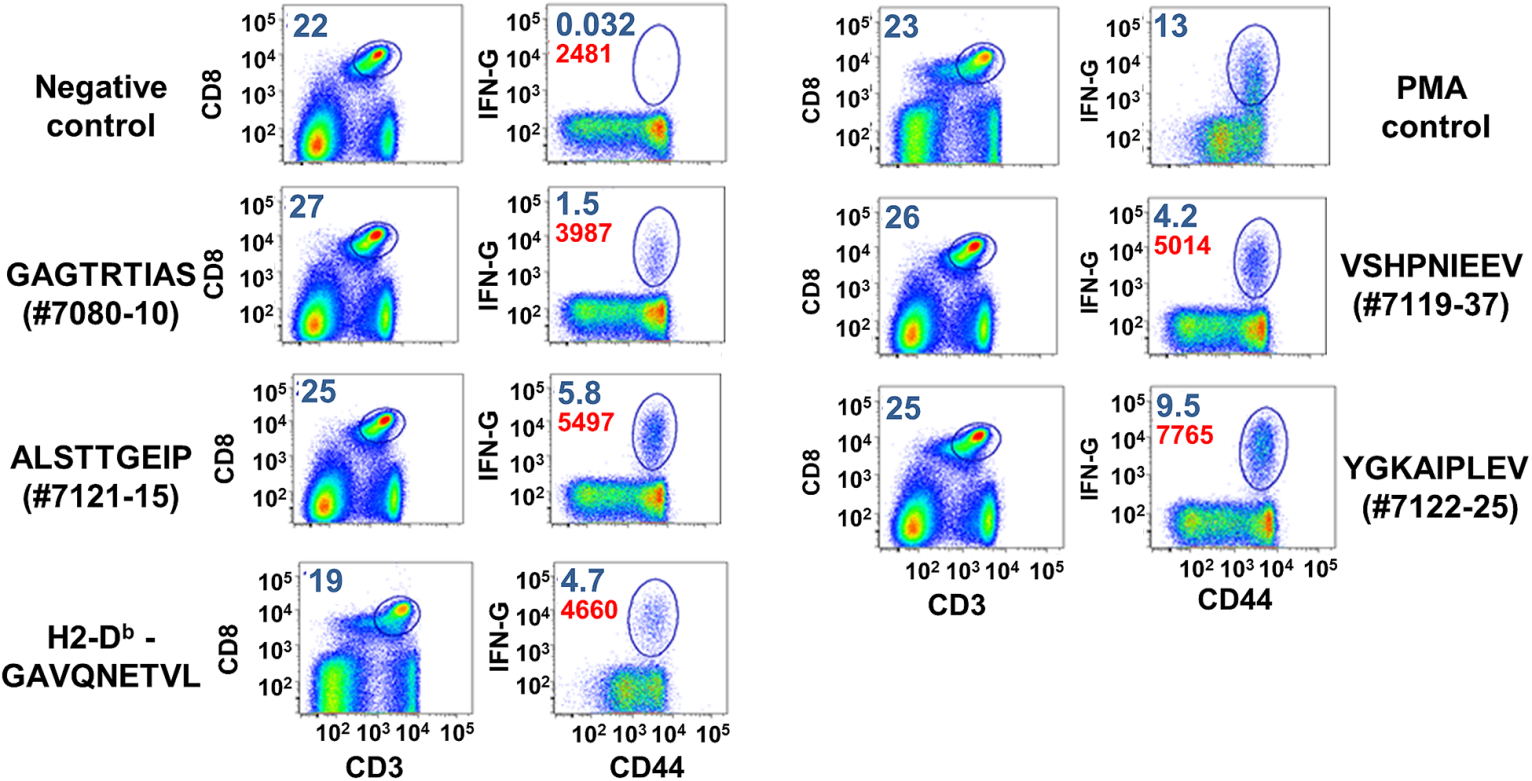
Identification of HCV-1a H77-NS3 CD8+ epitopes in the H2-b mouse system. Representative flow plots of IFN-γ positive CD8+ T-cells from the spleens of mice immunized with Ad-5-NS3/4A. Cells from immunized mice were isolated and stimulated with 0.1 μg/well of each peptide for 5 hours. The sequences GAGTRTIAS (aa1084-1092), ALSTTGEIP (aa1366-1374), YGKAIPLEV (aa1376-1384) and VSHPNIEEV (1357–1365) were 9 mers identified as CD8+ T-cell epitopes within the HCV NS3. Amino acid numbering is according to the H77 GT1a polyprotein sequence. Blue numbers on the plots are percentages gated on live CD8+ T-cells (right panels) and CD8+ T-cells positive for CD44 and IFN-γ. Red numbers on the plots represent mean fluorescence intensity for CD8+/CD44+/IFN-γ+ cells. Negative control represents mock stimulated cells. These experiments were repeated at least 5 times.

There exists a commercially available pentamer for the HCV-NS3 protein which includes the GAVQNEVTL sequence located at aa1629-1637 of the HCV polyprotein. In order to obtain a broader range of peptides for testing responses to HCV-NS3 in the B6 mice we performed an analysis to identify new epitopes using a GAVQNEVTL peptide as the positive control. Peptides representing GT1a-H77 spanning the entire HCV NS3 sequence were synthesized as 18mers overlapping by 8 amino acids (Mimotopes, Clayton, Australia). Splenocytes from Ad5-NS3 and VV-NS3 immunized mice were isolated and stimulated with pools of 9–10 NS3 peptides per reaction and screened for IFN-γ cytokine production by flow cytometry. After the initial screening, peptides from positive pools were individually used for repeated stimulation of IFN-γ production to identify the specific epitope sequence. From this analysis four 18mer peptides were identified that yielded specific IFN-γ responses in CD8+ T-cells in addition to the GAVQNETVL sequence: #7080- WTVYHGAGTRTIASPKGP (aa1079-1096), #7119 –PGSVTVSHPNIEEVALST (aa1352-1369), #7121 –ALSTTGEIPFYGKAIPLE (aa1366-1383), and #7122 –IPFYGKAIPLEVIKGGRH (aa1373-1390) (data not shown). A library of 9mer and 15mer peptides was then synthesized for re-screening and finer mapping of the specific epitope sequences. Four 9mer peptides located within these 18mers were identified as novel CD8+ T-cell epitopes GAGTRTIAS (#7080–10), VSHPNIEEV (#7119–37), ALSTTGEIP (#7121–15), and YGKAIPLEV (#7122–25) (Fig 1). These peptides or the original 18mers were used for stimulation of splenocytes as indicated below.

### Ad5-NS3 boosting induces a higher magnitude of CD8+ T-cell responses

**Fig 2:**
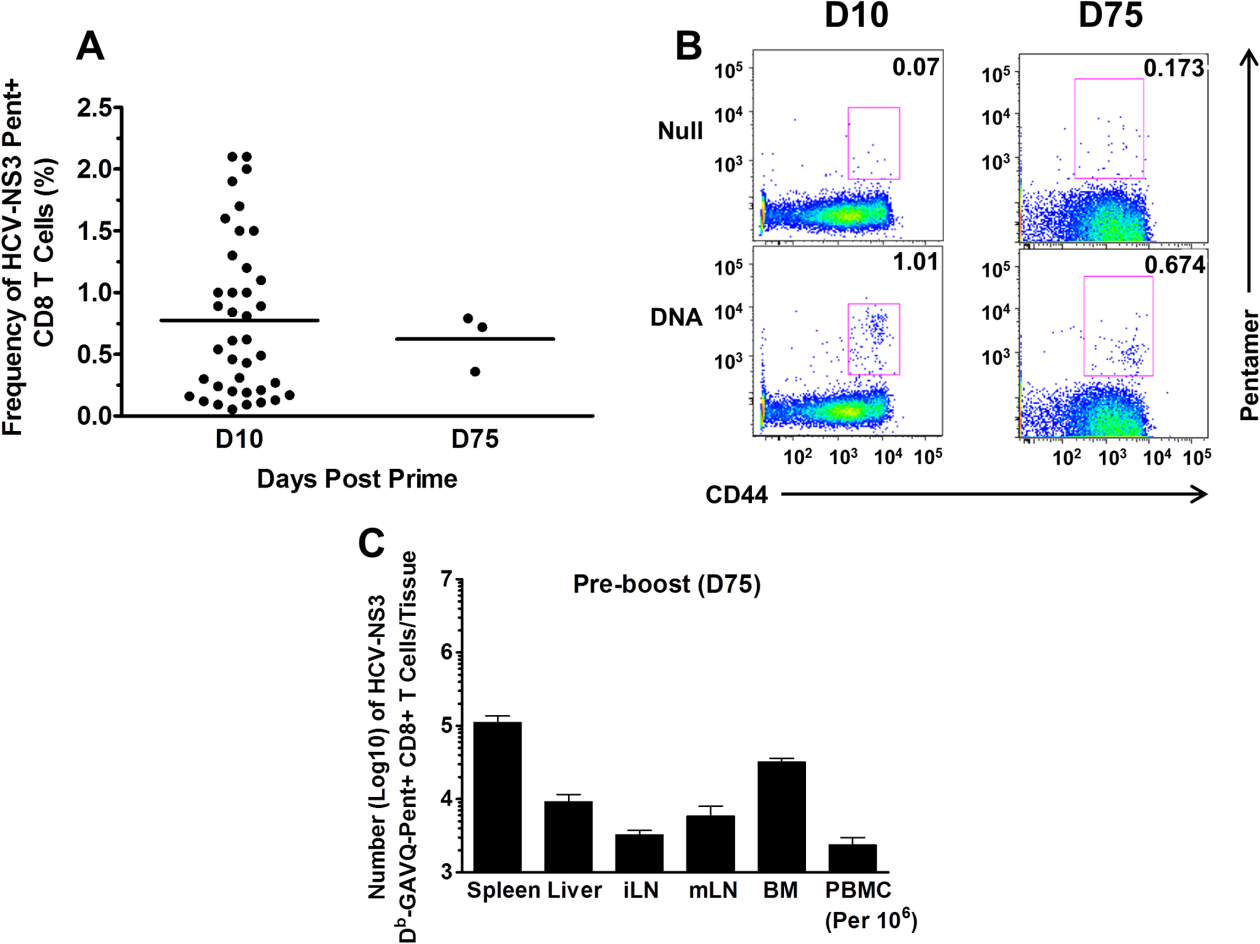
Magnitude of CD8+ T-cell responses post immunization. (A) HCV-NS3 pentamer positive (Pent+) CD8+ T-cells present in the blood of mice after priming with 100μg of plasmid DNA containing the full length HCV-NS3/4A gene insert at D10 (n = 39) and D75 (n = 3) post DNA immunization. Horizontal bars represent the mean frequency in each group (B) Representative flow plots of CD44+/Pent+ cells isolated from the blood of DNA immunized mice at D10 and D75 Black numbers on the plots are percentages positive for CD44 and pentamer after gating on CD8+ and CD3+ cells. (C) Numbers of Pent+ CD8+ T-cells accumulated in both lymphoid and non-lymphoid tissues of immunized mice at D75 post DNA-NS3/4A prime. PBMCs were enumerated based on per million cells. n = 3 mice per group.

To characterize the immune response elicited post vaccination, we first primed groups of B6 mice with 100 μg/mouse of plasmid DNA expressing the HCV-NS3/4A delivered intramuscularly via the hind quadriceps muscles. At D10 post prime, HCV-NS3 MHC class I H2D^b^ pentamer+ (GAVQ) CD8+ T-cells were detectable in the blood of immunized mice at between 0.05–2.1% of total CD8+ T-cells (mean of 0.8% for n = 39) (Fig 2A). These values corresponded to 1 to 38-fold over the frequencies observed in naïve mice tested in the same assay (Fig 2B). The mean frequencies declined to about 0.5% by D75 post prime (n = 3); this corresponded to a 2 to 4-fold increase over naïve mice tested in the same assay (Fig 2A and 2B). We also analyzed tissues of immunized mice at D75 to assess the numbers of NS3-specific CD8+ T-cells maintained in both the lymphoid and non-lymphoid compartments and found the highest number of cells were maintained in the spleen (~100,000 cells) and bone marrow (BM ~90,000 cells), with ~10^3^ to 10^4^ cells in the liver, inguinal lymph nodes, mesenteric lymph nodes and blood (Fig 2C).

**Fig 3:**
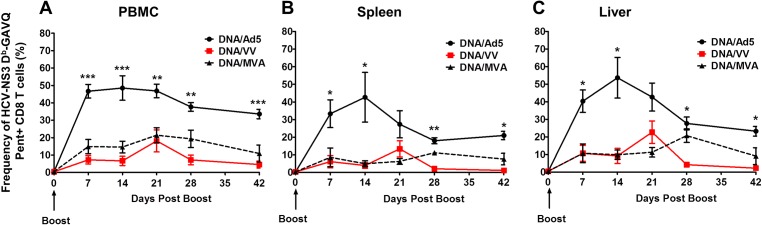
Kinetics of antigen-specific secondary effector CD8+ T-cell expansion of immunized mice after boosting. Mice were primed with DNA and boosted with Ad5-NS3/4A (Ad5) (10^9^ vp), VV-NS3/4A (VV) (2 x 10^6^ pfu) and MVA-NS3/4A (MVA) (10^8^ IU). (A) Blood (n = 7). (B) Spleen (n = 3–4 mice per group) and (C) Liver (n = 3–4 mice per group). Error bars represent standard error of the mean. Asterisks represent significance analyses following post hoc Bonferroni testing between Ad5 and VV or Ad5 and MVA immunized mice. * = p value <0.05; ** = p value<0.01, *** = p value <0.0001. Where different p values were obtained for VV and MVA compared to Ad5 the lower value is represented.

Mice were then boosted with 10^9^ vp of Ad5-NS3/4A (Ad5), 2x10^6^ pfu of VV-NS3/4A (VV) or 10^8^ IU of MVA-NS3/4A (MVA) at D85 post prime and the kinetics of secondary antigen-specific CD8+ T-cell responses in the blood and tissues were assessed using MHC Class I pentamer (H2D^b^-GAVQ) binding assays. Comparisons between the peak responses for the three vaccine vectors indicated that Ad5 was significantly more immunogenic than MVA or VV, both of which showed a similar magnitude of responses (S1 Fig). Tremendous expansion of the pentamer+ CD8+ T-cells was observed in the blood, spleen and liver of mice boosted with Ad5 (Fig 3A–3C). At D7 post boost (D92 post immunization), the magnitude of pentamer+ CD8+ T-cells in the blood after Ad5 administration was ~4-fold higher than boosting with MVA (p<0.0001) and ~8-fold higher than boosting with VV (p<0.0001) (Fig 3A). Similar differences in the magnitude of responses were also found in the spleens and livers of immunized mice (Fig 3B and 3C). The peak response to the Ad5 boost was at D14 in the blood, spleen and liver (Fig 3A–3C) while peak responses for VV were observed at D21 post boost in all tissues analyzed. Interestingly, boosting with MVA showed a peak response in the blood at D21 but delayed responses in the spleen and liver (D28 post boost) compared to Ad5 and VV (Fig 3A–3C).

**Fig 4:**
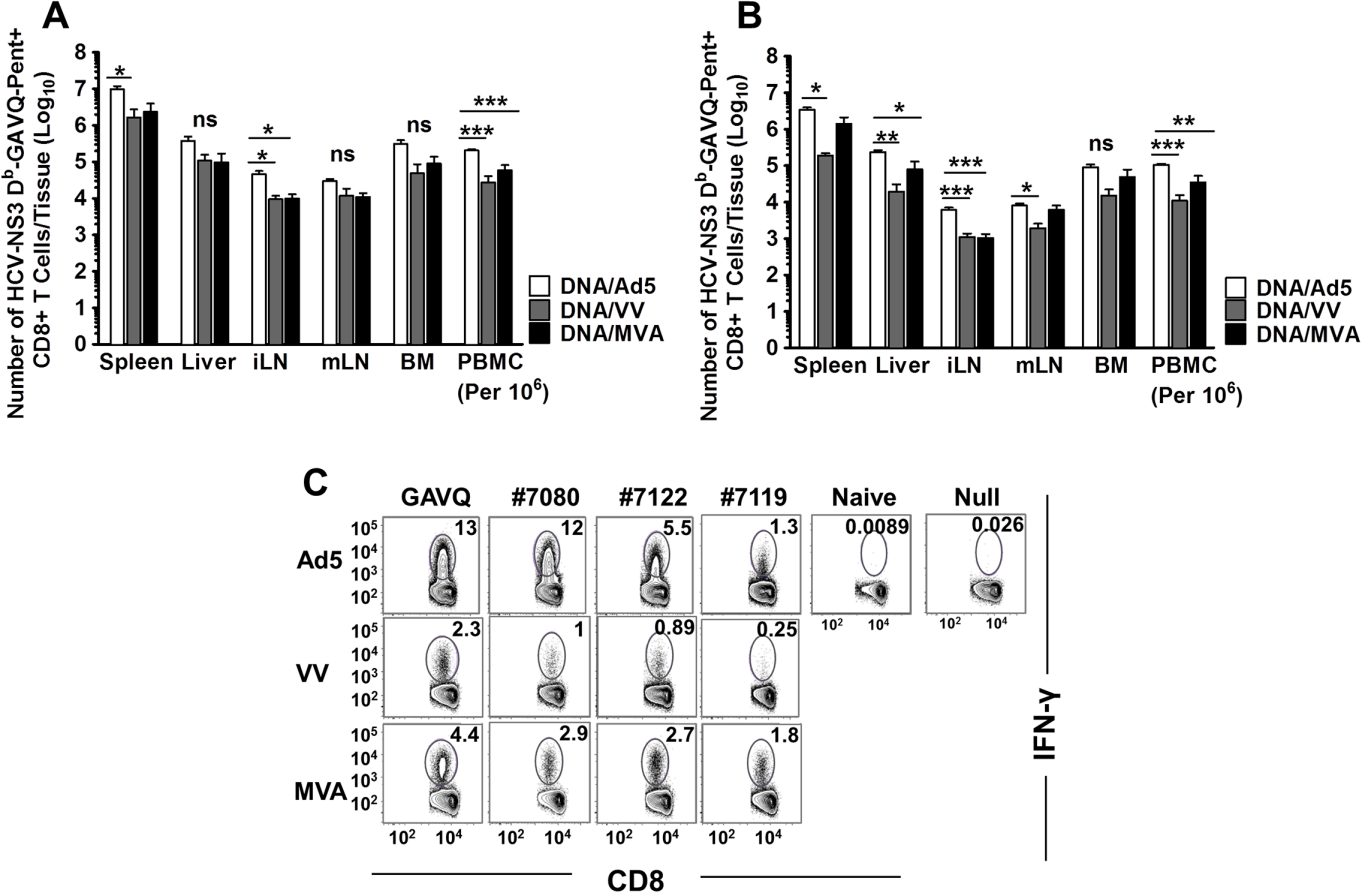
Numbers of pentamer+ CD8+ T-cells present in tissue compartments. (A) D7 post boost, n = 3–4 mice per group. (B) D42 post boost, n = 3–4 mice per group. (C) Breadth of antigen-specific CD8+ T-cell response shown as IFN-γ production upon stimulation with peptides representing four different CD8 T-cell epitopes of the HCV-NS3 antigen at D7 post boost. n = 3–4 mice per group Error bars represent standard error of the mean. ns = no significance between the means determined by one-way ANOVA. Asterisks represent significance analyses following post hoc Bonferroni testing between Ad5 and VV or Ad5 and MVA immunized mice. * = p value <0.05; ** = p value<0.01, *** = p value <0.0001.

T-cell expansion at D7 post boost in all tissues analyzed was higher in Ad5 immunized mice compared to MVA or VV immunized mice (Fig 4A). These differences were found to be statistically significant in the spleen, inguinal lymph nodes, and PBMC. Contraction of NS3-specific CD8+ T-cells was observed by D42 post boost (D127 post immunization) in the tissues amongst all vaccination regimens (Figs 3 and 4B). However, Ad5 boosted mice showed a sustained level of pentamer+/CD8+ T-cells in blood and tissue compartments compared to VV and MVA (Figs 3 and 4B).

In order to assess the breadth of CD8+ T-cell responses we stimulated splenocytes from immunized mice at D7 post boost with NS3 peptides representing different CD8+ T-cell epitopes and stained for IFN-γ. Our results showed that boosting with Ad5, MVA and VV generated antigen-specific CD8+ T-cells that are targeted towards the dominant GAVQ epitope and sub-dominant #7080, #7122, and #7119 epitopes in a hierarchical manner (Fig 4C) with the strongest responses being maintained in Ad5 immunized mice. These data show that all three vaccines are able to elicit a broad CD8+ T-cell response towards various epitopes of the HCV-NS3 protein but that Ad5 is significantly more immunogenic than MVA and VV in boosting antigen-specific responses post DNA priming in mice.

### Ad5 boosting induces qualitatively different memory CD8+ T-cells

To determine the phenotypes of the NS3-specific CD8+ T-cells, we analyzed several phenotypic markers that are routinely used to differentiate effector and memory CD8+ T-cells. We examined the quality of the vaccine-induced memory CD8+ T-cells by studying the expression kinetics of CD127, a hallmark feature of long-lived memory T-cells, Bcl-2 (anti-apoptotic molecule), CD62L (lymphoid trafficking marker), CD44 –a cell adhesion receptor, which is essential for cell trafficking and highly expressed on activated and memory T- and B-cells, CD27 –a costimulatory molecule for T lymphocytes, CXCR3 –a chemokine receptor which is highly expressed in activated T, B and NK cells for chemotaxis, and Ly6C –a GPI-linked surface antigen found on most cells and which is also a memory marker for CD8+ T-cells.

**Fig 5:**
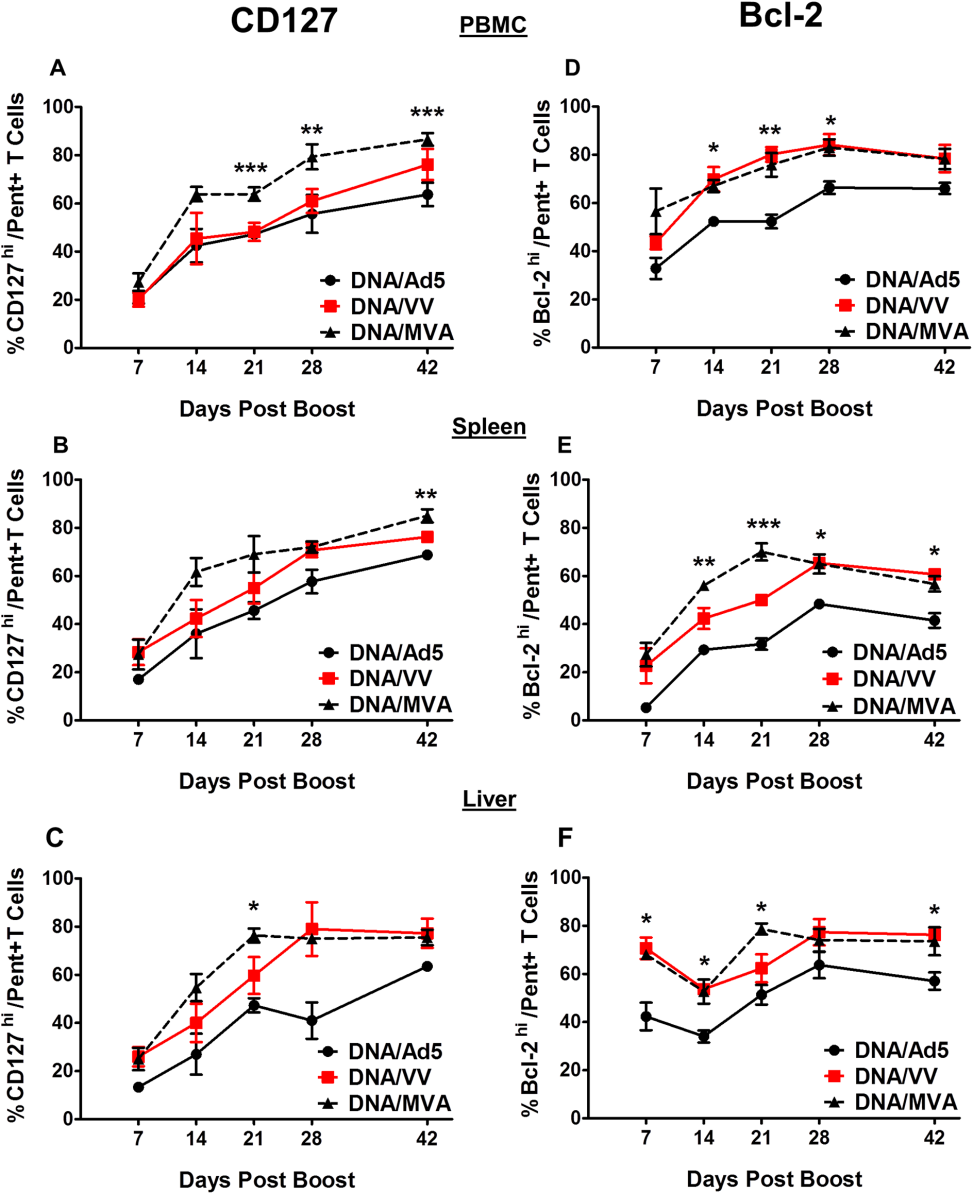
Phenotypes of GAVQ Pent+ CD8+ T-cells in the tissues of immunized mice after boosting with Ad5-, VV- or MVA-NS3/4A vectors. Kinetics of CD127 expression in (A) blood; (B) spleen, and (C) liver. Kinetics of Bcl-2 expression in (D) blood, (E) spleen, and (F) liver, Blood n = 7 mice per group, spleen and liver n = 3–4 mice per group. Error bars represent standard error of the mean. For clarity asterisks represent significance analyses following post hoc Bonferroni testing between Ad5 and MVA immunized mice. * = p value <0.05; ** = p value<0.01, *** = p value <0.0001.

The kinetics of most markers when analyzed as a frequency within total CD8+ cells were similar to those seen for pentamer positive cells with higher frequencies seen in the Ad5 boosted group (data not shown). However, for some markers this was not the case which prompted us to analyze the frequency of cells within the pentamer positive group alone. We observed similar frequencies of pentamer positive cells expressing CD27, CD44, CXCR3, CD62L and Ly6C in the blood and tissues of all boosted mice (data not shown). However, the expression kinetics of CD127 and Bcl-2 in the blood, spleens and livers of pentamer positive cells from Ad5 boosted mice were significantly lower compared to the VV and MVA boosted mice (Fig 5). Although we observed statistically significant differences between Ad5 and VV immunized mice at several time points, for clarity, significant differences in Fig 5 are only shown for Ad5 compared to MVA immunized mice. At Day 0, data from all immunized mice showed high expression (>90% of cells positive) for both CD127 and Bcl-2 on the small numbers of pentamer positive cells detected (data not shown). At D7 post boost all groups of immunized mice displayed down-regulated expression of CD127 and Bcl-2 on pentamer+ cells in the blood, spleen and liver (Fig 5A–5F). These two markers were observed to re-express at D14 post boost but with a consistently lower level of expression in the Ad5 boosted mice than for the other 2 groups. In the blood, we observed that Ad5 boosted mice re-expressed significantly lower levels of pentamer+ CD127 throughout the post challenge period (Fig 5A) compared to MVA and VV boosted mice. Statistically significant differences in the expression of CD127 in the spleen at D42 post boost (Fig 5B) and liver at D21, (Fig 5C) were also observed. Similarly, statistically significant differences in the expression of Bcl-2 were noted in the blood, spleen and liver throughout the post challenge period (Fig 5D–5F). Notably, these differences were still detected at the memory time point in the spleen and liver (D42 post boost). These data suggest that although Ad5 boosting after DNA priming generated significantly more HCV-specific CD8+ T-cells, this population in the Ad5 boosted mice displayed differential expression of memory cell markers with a lower proportion of optimal memory phenotypes of CD8+ T-cells induced.

### Ad5 induced NS3-Specific CD8+ T-cells are less multifunctional than those induced by VV and MVA

**Fig 6:**
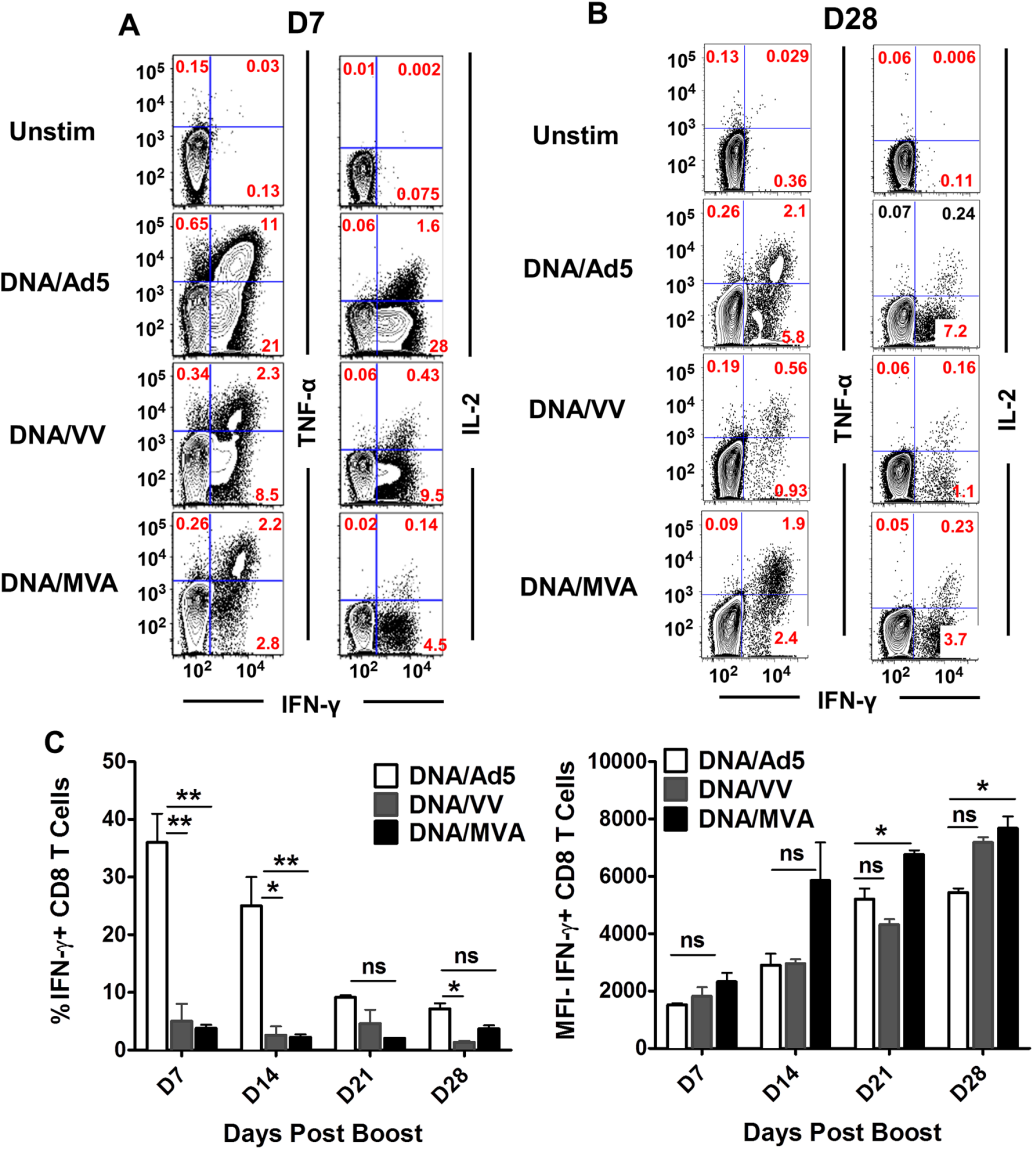
Functional characteristics of cytokine production capacities of HCV-NS3 antigen-specific CD8+ T-cells post boost with Ad5-, VV- or MVA-NS3/4A vectors. Freshly isolated splenocytes were stimulated with 0.1 ug/well of GAVQ peptide per 1.5 million cells and cultured in 10% FBS plus RPMI medium at 37°C for 5h. (A) Representative flow plots of intracellular staining of IFN-γ, TNF-α, and IL-2 in stimulated and unstimulated effector CD8+ T-cells (D7 post boost) and (B) memory precursor CD8+ T-cells (D28 post boost). Left panels show IFN-γ+/TNF-α+ co-producers and right panels show IFN-γ+/IL-2+ co-producers. Numbers in plots are representative percentages of cytokine producers. n = 3–4 mice per group. (C) Frequency (left) and mean fluorescence intensity (MFI, right) of IFN-γ producing CD8+ T-cells stimulated by GAVQpeptide. Error bars represent standard error of the mean. Asterisks represent significance analyses following post hoc Bonferroni testing between Ad5 and VV or Ad5 and MVA immunized mice. * = p value <0.05; ** = p value<0.01, *** = p value <0.0001.

Primed CD8+ T-cells produce several cytokines in the infected microenvironment that are required for the continued activation, expansion and cytolytic potential of T-cells [26]. In these studies, we examined the IL-2, IFN-γ and TNF-α cytokine production capacity of CD8+ T-cells after brief restimulation with antigen. Splenocytes from immunized mice at D7 and D28 post boost were isolated and stimulated with GAVQ peptide for five hours to induce production of intracellular cytokines. The frequencies of IFN-γ+/TNF-α+ and IFN-γ+/IL-2+ co-producing CD8+ T-cells were consistently higher in the Ad5 boosted mice at D7 post boost (Fig 6A). However, at D28 the MVA boosted mice appear to maintain the frequency of double cytokine producing cells while the levels in both Ad5 and VV boosted mice decreased to less than 50% of those seen at D7 (Fig 6B). In order to more closely compare the functional abilities of cells from the three vaccinated groups, we compared the frequencies of IFN-γ-producing CD8+ T-cells with the mean fluorescence intensity (MFI) of IFN-γ on a per cell basis after stimulation with the dominant GAVQ (Fig 6C) and sub-dominant #7080 and #7122 peptides (S2 Fig). We found that the Ad5 boosted group consistently generated a higher frequency of IFN-γ producing cells when compared to VV or MVA in response to stimulation by the three peptides throughout the course of vaccination from D7 to D42 (Fig 6C and S2 Fig, left panels). Interestingly, an inverse relationship was observed when compared with the MFI for IFN-γ. Although VV and MVA boosted mice have a lower frequency of IFN-γ producing CD8+ T-cells, the IFN-γ production capacity on a per cell basis was greater within these groups than for Ad5 boosted mice (Figs 6C and S2, right panels). Thus, although the Ad5 boost generated a high frequency of IFN-γ producing CD8+ T-cells, the fitness of these cells to produce copious amount of IFN-γ is not as high as that for cells boosted with VV or MVA.

**Fig 7:**
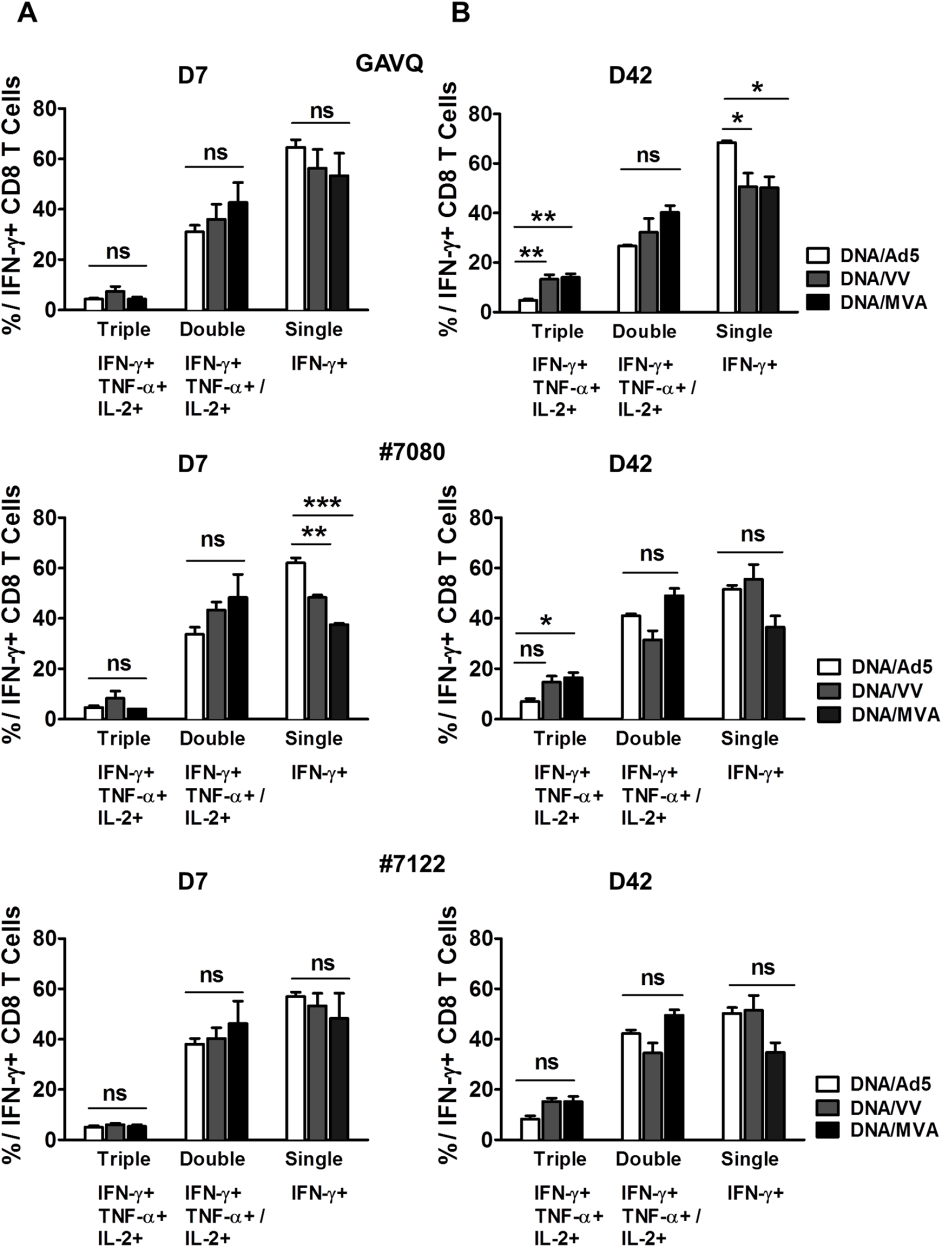
Frequencies of polyfunctional CD8+ T-cells post boost with Ad5-, VV- or MVA-NS3/4A vectors. Cells were stimulated with GAVQ, #7080 and #7122 at (A) D7 and (B) D42 post boost. Values are presented as single, double and triple cytokine-producing cells as a % of total IFN-γ positive cells. Asterisks represent significance analyses following post hoc Bonferroni testing between Ad5 and VV or Ad5 and MVA immunized mice. * = p value <0.05; ** = p value<0.01, *** = p value <0.0001. ns = non-significant, n = 3–4 mice per group. Error bars represent standard error of the mean.

We further compared the frequencies of CD8+ T-cells producing multiple cytokines simultaneously when stimulated with GAVQ, #7080 and #7122 peptides. Fig 7 shows the percentage of triple, double and single cytokine producers amongst all IFN-γ positive cells at D7 (Fig 7A) and D42 (Fig 7B) post boost. At D7, there was a trend for Ad5 boosted mice to produce fewer triple and double cytokine producing cells (Fig 7A, left panels) although these differences were not found to be statistically significant. At D42 (memory) Ad5 boosted mice also produced lower frequencies of triple (IFN-γ+ TNF-α+ IL-2+) and double (IFN-γ+ TNF-α+ or IFN-γ+ IL-2+) cytokine producers specific for all three peptides and the differences were found to be statistically significant for triple producers specific for the immunodominant peptide GAVQ compared to both VV and MVA boosted mice (Fig 7B). These results demonstrate that although Ad5 boost generated a high frequency of IFN-γ producing cells, these cells are less multifunctional than those induced by VV and, in particular, by MVA.

### Higher proliferation potential and cytolytic activity of MVA-induced antigen-specific CD8+ T-cells during direct challenge and recall of memory CD8+ T-cells

Thus far, our studies showed that VV and MVA boosted mice generated HCV-NS3-specific memory CD8+ T-cells that appear to be more long-lived and polyfunctional in producing IFN-γ, TNF-α and IL-2 compared to Ad5 boosted mice. To determine if these differences influence the proliferation potential during recall of the secondary memory CD8+ T-cell response, we performed a surrogate challenge of immunized mice at D85 post boost (memory) with 10^5^ pfu of murine γ-herpes virus-68 (MHV-68) expressing the HCV NS3 (MHV-68-NS3) [27]. To compare responses between secondary memory and primary immunization expansion of CD8+ T-cells, we also challenged naïve mice with the same dose of MHV-68-NS3. We harvested the blood and spleens of mice at D2, D6, D9, D13 and D16 post challenge for comparative studies using the NS3-specific pentamer (GAVQ). We also assessed virus titers in the spleens of mice at D2, D4, D6, D9, D12 and D14.

MHV-68-NS3 titers peaked in naïve mice at D4 post challenge (~3x10^3^ pfu/spleen) (data not shown). However, we were unable to quantify virus in the spleens of any immunized mice challenged with MHV-68-NS3. Previous data showed that immunization with MVA-OVA resulted in significant reductions in intrasplenic virus titers following challenge with MHV-68-OVA [29], to levels below the limit of detection. We concluded that the immune responses induced by all three vaccine regimens effectively controlled the MHV-68-NS3 replication. Therefore, for these studies the MHV-68-NS3 challenge served as a means to examine the characteristics of the recall response in immunized mice.

**Fig 8:**
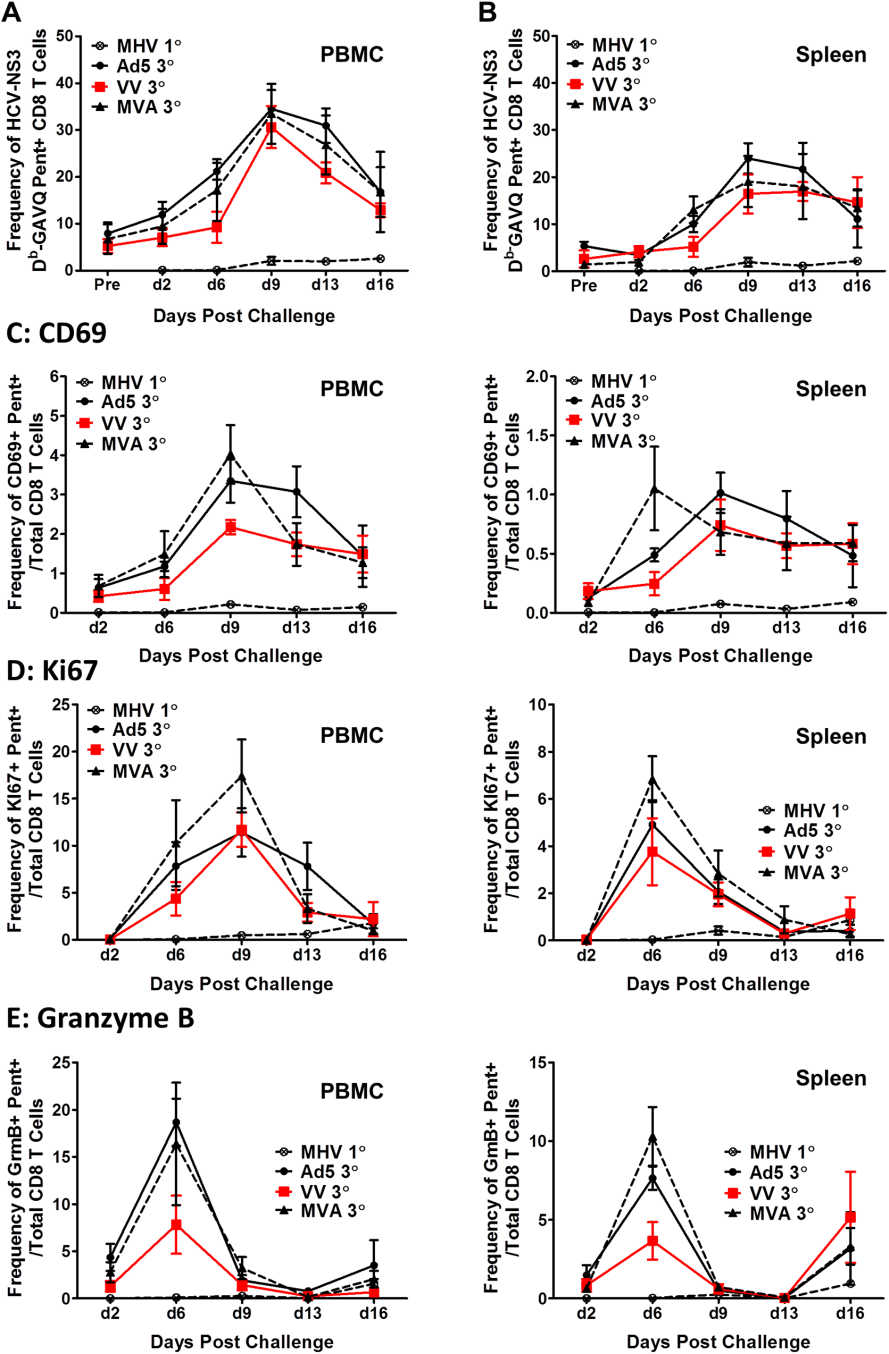
Recall expansion of HCV-NS3 antigen-specific CD8+ T-cells upon challenge with MHV-68-NS3 in immunized mice. Expansion of tertiary effector CD8+ T-cells of immunized memory mice (3°) in comparison with primary effector expansion in naïve mice (MHV 1°) challenged with MHV-68-NS3 in (A) blood and (B) spleen. (C) Expression of the early activation marker CD69 on pentamer+/CD8+ T-cells upon challenge with MHV-68-NS3 in the blood and spleen of immunized mice. (D) proliferation marker KI67 (E) cytolytic activity marker Granzyme B. Error bars represent standard error of the mean (n = 3–5 mice).

At D2 post challenge, pentamer+ CD8+ T-cells were detected with minimal expansion in both the blood and spleens (Fig 8A and 8B). However, expansion of the NS3 antigen-specific CD8+ T-cells increased to a similar degree at D6 in all immunized mice, to a peak of ~30% in the blood (Fig 8A) and ~20% in the spleen (Fig 8B) by D9 post challenge. As expected, in all analyses we observed minimal expansion of NS3-specific CD8+ T-cells during primary infection with MHV-68-NS3 (MHV 1°) in all tissues analyzed (Fig 8). We next compared the early activation marker CD69, the proliferation marker Ki67, and Granzyme B (GmB) (representative of cytolytic activities of activated CD8 T-cells) in the blood and spleens of challenged mice.

We found that activation of CD69 peaked at D9 post challenge in the blood for all three vaccination regimens (Fig 8C, left panel) while in the spleen the peak activation was at D6 for the MVA vaccinated group and at D9 for the VV and Ad5 group (Fig 8C, right panel). Proliferation of the expanded CD8+ T-cells, as assessed by Ki67, showed a peak response at D9 for the all boosted groups (Fig 8D, left panel) while in the spleen all groups showed peak proliferation at D6 post challenge (Fig 8D, right panel). In contrast, GmB production peaked at D6 post challenge in the blood and spleens of all immunized mice (Fig 8E). Although the kinetics of NS3-specific T-cell expansion following challenge with MHV-68-NS3 was similar amongst all immunized groups of mice, we observed consistently higher levels of CD8+/pentamer+ cells that were also positive for CD69+, Ki67+ and GmB+ in the spleens of MVA immunized mice.

**Fig 9:**
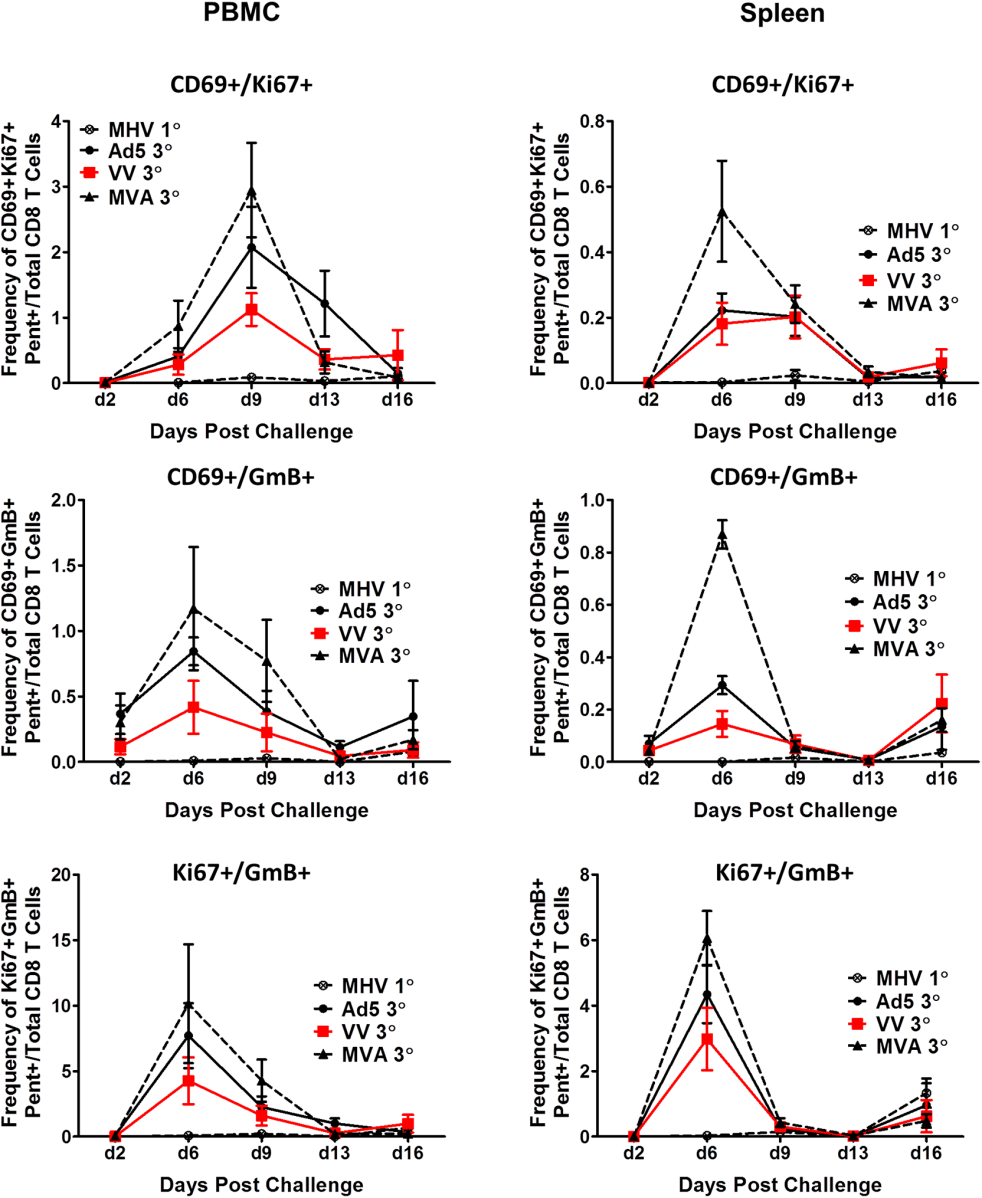
Recall expansion of HCV-NS3 antigen-specific CD8+ T-cells expressing combinations of markers for proliferation (CD69), activation (Ki67) or cytolytic activity (GmB) in the blood and spleens upon challenge with MHV-68-NS3 in immunized mice. Error bars represent standard error of the mean, n = 3–5 mice per group.

We have previously shown that CD8+ T-cells that are both proliferating and activated are associated with clearance in immune primed chimpanzees following infection with HCV [20]. Therefore, we analyzed the frequencies of cells expressing combinations of the CD69, Ki67, and GmB markers (CD69+/Ki67+, CD69+/GmB+,and Ki67+/GmB+ cells) in the blood and spleens of immunized mice following challenge. We found that mice immunized with MVA-NS3 had higher frequencies of cells expressing CD69 in combination with Ki67 or GmB at the peak time points during the post challenge period in the blood and spleens (Fig 9). These data further indicate that the secondary memory cells recalled after challenge with virus expressing the HCV NS3 protein are qualitatively different in the Ad5 immunized mice and suggest that NS3-specific memory T-cells induced by MVA have higher proliferative and cytolytic capacities.

## Discussion

HCV persists in the majority of infected individuals but it is cleared in a small proportion of cases, which results in memory responses that lead to early and rapid control of the virus upon subsequent reinfection [30, 31]. Despite this development of natural immunity to the virus following clearance, the development of vaccines to prevent HCV infection has proved challenging. Phase I clinical trials have shown that immune responses to the virus can be induced with recombinant proteins or viral vectors [32, 33].

In this study, we compared three viral vectors (Ad5, VV and MVA) commonly used for the induction of T-cell responses against transgenes and have shown significant differences in the memory CD8+ T-cells induced against the HCV NS3 protein which could prove pivotal in the control and clearance of HCV following infection. Our data show that boosting with Ad5 following DNA priming leads to rapid expansion of NS3-specific CD8+ T-cells in B6 mice resulting in significantly higher frequencies than those obtained following VV or MVA boosting. These differences were maintained across different tissues (blood, spleen and liver) against multiple HCV-NS3 epitopes and throughout the follow up period post boost (Fig 2). However, when the T-cell responses were analyzed for specific memory cell markers, we found that Ad5 induced lower frequencies of CD8+ T-cells expressing the memory cell marker CD127 and fewer CD8+ T-cells expressing the anti-apoptotic molecule Bcl-2 in the blood, spleen, and liver compared to cells induced by VV and MVA. This difference was observed throughout the post boost follow-up period, and the differences were found to be significant (Fig 3). The T-cell phenotypes in the liver compartment could be particularly relevant for the control of HCV as this is the primary site of viral replication in the body. The quality of memory T-cells has been linked with the expression of CD127 [34] and Bcl-2; [35] thus despite high levels of NS3-specific T-cells induced by Ad5, a lower proportion of these cells appear to be associated with memory function. This conclusion was further confirmed when cytokine production and polyfunctionality were assessed in stimulated T-cells. There appeared to be a rapid expansion of cytokine producing cells following Ad5 boosting but this response also contracted rapidly compared to a steady maintenance of HCV-specific T-cells in mice boosted with VV and MVA. More importantly, the per cell production of IFN-γ was inferior in the Ad5 boosted group compared to the VV and MVA boosted groups as the numbers of polyfunctional CD8+ T-cells were significantly lower in the Ad5 boosted group at later time points as evidenced by the frequencies of triple and double cytokine-producing cells.

Overall, our data suggested that the memory cells induced by Ad5 were inferior to those induced by VV or MVA. In addition our data suggested that the memory cells induced following boosting with MVA were superior to those induced by both Ad5 and VV boost. The numbers of cells expressing CD127 and Bcl-2 were generally higher for MVA boosted mice in the blood, spleen, and liver, the per cell IFN-γ production was highest for CD8+ cells from mice boosted with MVA, and also the proportion of polyfunctional CD8+ NS3-specific cells was consistently higher in the MVA boosted mice. Following tertiary exposure to the HCV NS3 antigen, through challenge with the MHV-68-NS3 recombinant virus, we observed expansion of HCV-specific cells in response to infection in all immunized groups but MVA boosted mice showed consistently higher levels of cells expressing CD69, Ki67 or Granzyme B, markers that are commonly identified with functional T-cell activation in response to antigens. In addition, the MVA-immunized mice displayed higher levels of cells that were both activated and proliferating. We have previously shown that ineffective T-cell responses following immune priming in chimpanzees are associated with qualitative differences in memory T-cells and, specifically that proliferating, activated memory CD8+ T-cells are associated with clearance [20].

As expected all three vectors in this study elicited HCV-specific memory T-cells that were recalled upon exposure to virus expressing the NS3 antigen, indicating that all three vectors induced memory T-cell responses. However, the control of HCV replication and the progression to chronic infection appears to be a very subtle process that is not solely dependent on the presence or absence of immune responses. T-cell based chimpanzee vaccine studies have all induced HCV-specific immune responses, regardless of the immunogen used, and in all cases viral replication is controlled rapidly following challenge with HCV [18]. However, despite this early control, the outcome from challenge has still resulted in persistence of the virus in a large percentage of challenged animals [18]. Thus it appears that T-cell responses induced by vaccination have the ability to control viral replication immediately following challenge but cannot maintain the control; leading to long-term persistence. The maintenance of specific memory T-cells could be the critical difference between clearance of virus and persistence. The previous chimpanzee vaccine studies used all three vectors examined in our studies either alone or in combination with DNA priming; persistence and clearance occurred in immunized animals regardless of the vector used [18]. We previously assessed if the type of recombinant viral vector used to induce T-cells in these experimental vaccines impacted the outcome of infection after challenge but found the rate of persistence or clearance could not be associated with any particular vector [18]. The numbers of animals per group, different challenge doses of HCV and the inclusion of different antigens in the vaccines makes direct comparisons difficult. The majority of studies did not examine T-cell phenotypes or memory markers following immunization or challenge of animals, therefore conclusions on the role of T-cell memory in the success or failure of any one vaccine is challenging.

The differences we observed for NS3-specific CD8+ T-cell kinetics and memory phenotypes may partly be explained by the kinetics and magnitude of transgene expression by each of the viral vectors we studied. Levels and longevity of antigen can influence cytokine expression and stimulatory signals that lead to the differentiation and survival of memory T-cells. We found that the Ad5 vector used in our studies expresses higher levels of transgene than the VV and MVA vectors (data not shown) which may account for the rapid expansion of specific T-cells post boost with the Ad5 vector. However, other studies have shown that the levels of antigen expression by viral vectors post boost have a minimal effect on T-cell differentiation as the duration of expression is brief in all cases [36]. Conversely, the Ad5 vector used in our studies was replication incompetent while the VV vector was a replicating virus which would result in cell-to-cell spread and new viral infections suggesting that although the initial levels of antigen expressed by the Ad5 vector may be higher the expression of antigen by VV would continue for longer periods of time. Non-replicating MVA lacks many of the immune evasion factors VV uses to interfere with key innate immune responses of the host which may explain for the particular immunomodulatory capacity of MVA-based vaccines (for review see [37]). Native viral antigens expressed as part of the vectors during protein expression could equally account for the impact on T-cell differentiation and development, such as CD4+ responses to viral vector antigens. As has been previously reported memory CD8+ T-cell responses induced by different viral vectors exhibit different functional qualities [36]. We show here that differences are maintained during subsequent exposure of immunized mice to the HCV NS3 antigen following challenge with a surrogate virus and that the differences are associated with memory T-cell phenotypes shown to be important for HCV clearance. Overall, our data indicate that the magnitude of specific cells induced during immunization is not necessarily an indicator of quality and suggest that MVA or VV represent a better alternative for induction of memory T-cells against the HCV NS3 protein compared to Ad5; which could have a considerable impact on the development of vaccines against this agent.

## Supporting information

S1 Fig Magnitude of CD8+ T-cell responses post immunization. Representative flow plots of HCV-NS3 GAVQNEVTL (GAVQ) Pent+ CD8+ T-cell responses in the blood of mice after heterologous prime-boost immunization. Black numbers represent the percentage of CD8+/Pentamer+ cells.
(TIF)

S2 Fig IFN-γ production in by peptide stimulated CD8+ T-cells. Functional characteristics of cytokine production capacities of HCV-NS3 antigen-specific CD8+ T-cells post boost with Ad5-, VV- or MVA-NS3/4A vectors. Frequencies (left) and mean fluorescence intensity (MFI, right) of IFN-γ producing CD8+ T-cells stimulated by #7080 and #7122 peptides. #7122 data for D14 not shown. Error bars represent standard error of the mean. Asterisks represent significance analyses following post hoc Bonferroni testing between Ad5 and VV or Ad5 and MVA immunized mice. * = p value <0.05; ** = p value<0.01, *** = p value <0.0001. ns = no significant differences between the means assessed by one way ANOVA.
(TIF)
