# Supplementary figures and images for: Qualitative differences in cellular immunogenicity elicited by hepatitis C virus T-Cell vaccines employing prime-boost regimens
Source: PLoS One. 2017 Jul 21;12(7):e0181578. doi: 10.1371/journal.pone.0181578 (PMC5521799; doi:10.1371/journal.pone.0181578)

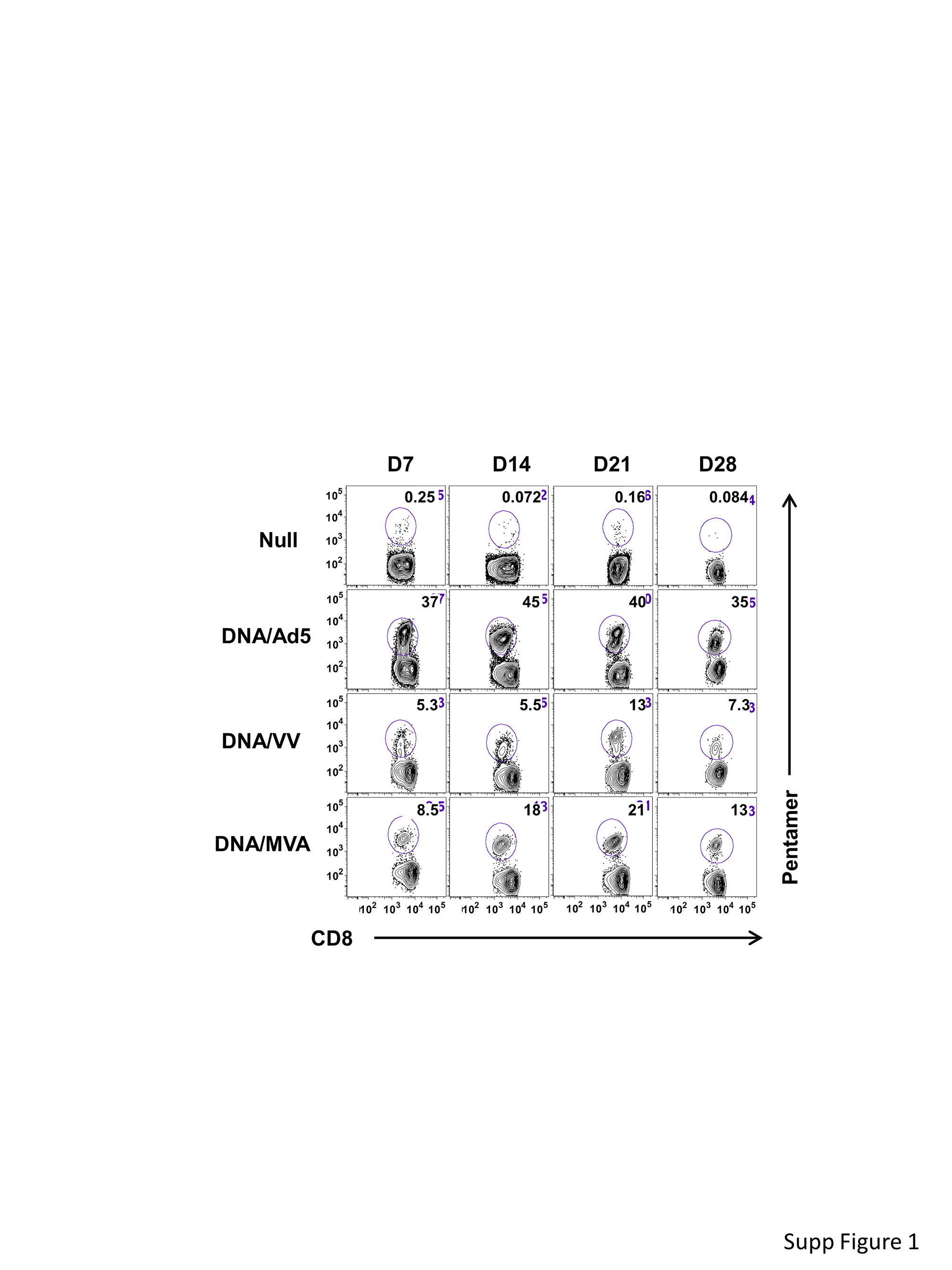

Supplement: S1 Fig — Representative flow plots of HCV-NS3 GAVQNEVTL (GAVQ) Pent+ CD8+ T-cell responses in the blood of mice after heterologous prime-boost immunization. Black numbers represent the percentage of CD8+/Pentamer+ cells. (TIF) [file pone.0181578.s001.tif]

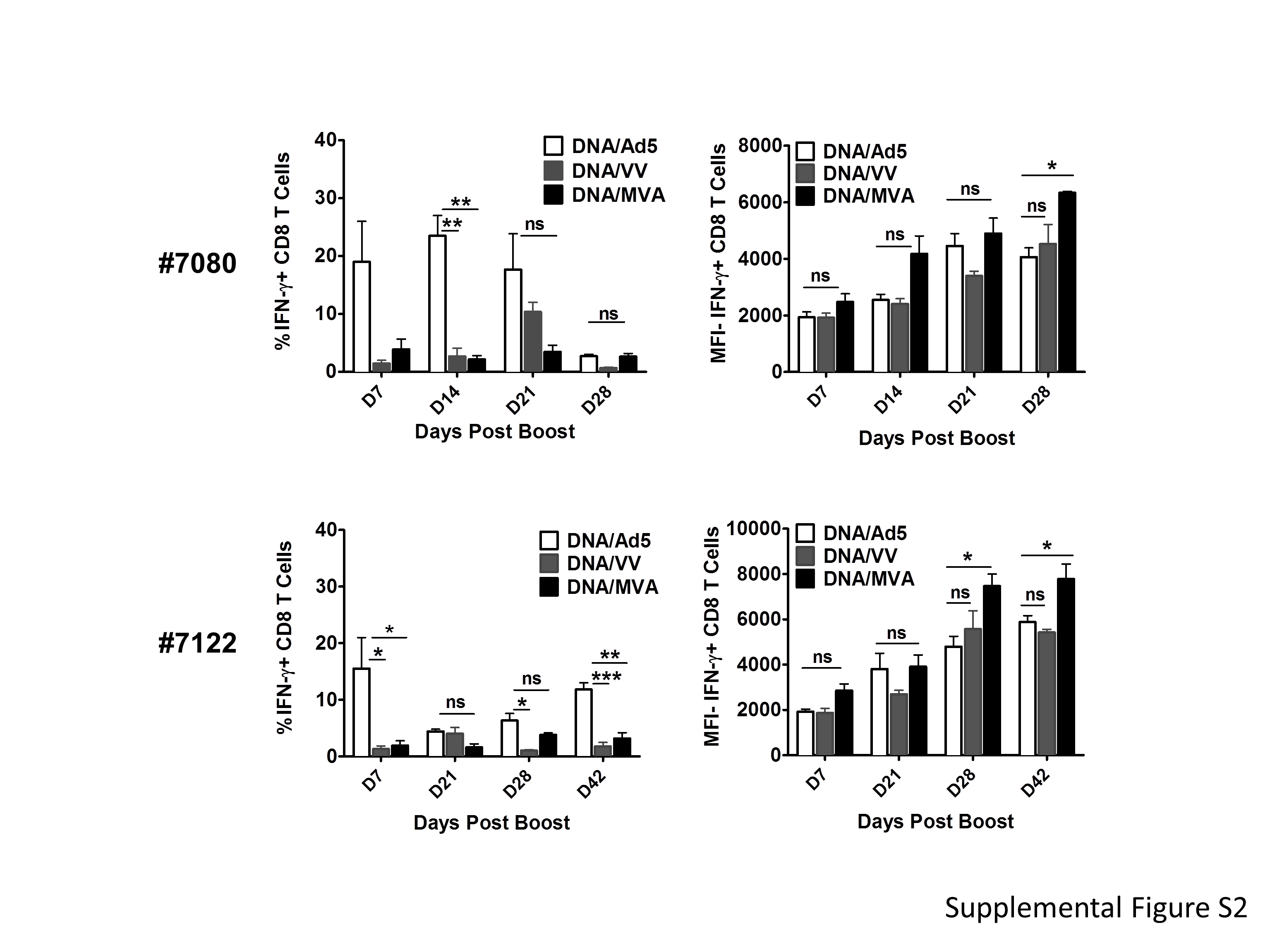

Supplement: S2 Fig — Functional characteristics of cytokine production capacities of HCV-NS3 antigen-specific CD8+ T-cells post boost with Ad5-, VV- or MVA-NS3/4A vectors. Frequencies (left) and mean fluorescence intensity (MFI, right) of IFN-γ producing CD8+ T-cells stimulated by #7080 and #7122 peptides. #7122 data for D14 not shown. Error bars represent standard error of the mean. Asterisks represent significance analyses following post hoc Bonferroni testing between Ad5 and VV or Ad5 and MVA immunized mice. * = p value <0.05; ** = p value<0.01, *** = p value <0.0001. ns = no significant differences between the means assessed by one way ANOVA. (TIF) [file pone.0181578.s002.tif]
